# A Study of Social Isolation, Multimorbidity and Multiple Role Demands among Middle-age Adults Based on the CLSA

**DOI:** 10.1093/geroni/igab046.2287

**Published:** 2021-12-17

**Authors:** Lun Li, Andrew Wister, Barbara Mitchell

**Affiliations:** 1 University of Simon Fraser, Vancouver, British Columbia, Canada; 2 Simon Fraser University, Vancouver, British Columbia, Canada

## Abstract

Our understanding of the influence of concurrent multiple social and family roles on social isolation among the middle-aged generation remains limited. Given the increasing complexity of parenting, caregiving and working patterns over recent decades in many countries, and greater concern of multimorbidity in mid-life, this study examines the longitudinal effects of these contexts on social isolation among middle-aged persons. We apply Linear Mixed Models to analyze a sub-sample of 29,847 middle-aged (aged 45 to 64) participants drawn from the Baseline and Follow-up 1 waves of the Canadian Longitudinal Study on Aging. Separated analyses were conducted for participants with or without multimorbidity in order to identify patterns across these groups. Both middle-aged participants with and without multimorbidity experienced greater social isolation over time. Among participants without multimorbidity, holding multiple roles serves as a protective function to prevent social isolation over time. Among participants with multimorbidity, the parenting role remain as a protective factor; however, the caregiving role increases the risk of social isolation over time. This study confirms several life-course transitions from middle age to older age, including increased risk of social isolation and caregiving demand, and decreased parenting and working involvement. Different associations were uncovered among middle-aged persons occupying multiple roles on social isolation with and without multimorbidity over time. The findings emphasize the necessity to study multimorbidity as a salient contextual factor, and to provide enhanced support to multimorbid middle-aged individuals with increasing family caregiving demands.

